# Predicting health crises from early warning signs in patient medical records

**DOI:** 10.1038/s41598-022-23900-8

**Published:** 2022-11-10

**Authors:** Selin Gumustop, Sebastian Gallo-Bernal, Fionnuala McPeake, Daniel Briggs, Michael S. Gee, Oleg S. Pianykh

**Affiliations:** 1 School of Medicine, Tufts University, Boston, MA USA; 2 Department of Radiology, Massachusetts General Hospital, 55 Fruit St, Boston, MA 02114 USA; 3 Harvard Medical School, Boston, MA USA

**Keywords:** Disease prevention, Public health, Risk factors

## Abstract

The COVID-19 global pandemic has caused unprecedented worldwide changes in healthcare delivery. While containment and mitigation approaches have been intensified, the progressive increase in the number of cases has overwhelmed health systems globally, highlighting the need for anticipation and prediction to be the basis of an efficient response system. This study demonstrates the role of population health metrics as early warning signs of future health crises. We retrospectively collected data from the emergency department of a large academic hospital in the northeastern United States from 01/01/2019 to 08/07/2021. A total of 377,694 patient records and 303 features were included for analysis. Departing from a multivariate artificial intelligence (AI) model initially developed to predict the risk of high-flow oxygen therapy or mechanical ventilation requirement during the COVID-19 pandemic, a total of 19 original variables and eight engineered features showing to be most predictive of the outcome were selected for further analysis. The temporal trends of the selected variables before and during the pandemic were characterized to determine their potential roles as early warning signs of future health crises. Temporal analysis of the individual variables included in the high-flow oxygen model showed that at a population level, the respiratory rate, temperature, low oxygen saturation, number of diagnoses during the first encounter, heart rate, BMI, age, sex, and neutrophil percentage demonstrated observable and traceable changes eight weeks before the first COVID-19 public health emergency declaration. Additionally, the engineered rule-based features built from the original variables also exhibited a pre-pandemic surge that preceded the first pandemic wave in spring 2020. Our findings suggest that the changes in routine population health metrics may serve as early warnings of future crises. This justifies the development of patient health surveillance systems, that can continuously monitor population health features, and alarm of new approaching public health crises before they become devastating.

## Introduction

COVID-19, a highly infectious disease caused by the Severe Acute Respiratory Syndrome Coronavirus 2 (SARS-CoV-2), has resulted in one of the most severe and extensive disease outbreaks in human history, devastating both the economy and health systems worldwide. Since the pandemic's beginning, the World Health Organization (WHO) has reported more than 6 million deaths globally due to COVID-19^1^.

While the first cases of COVID-19 in the United States were officially reported back in January and February of 2020^2^, strict federal public health guidelines in the United States were only implemented in the middle of March 2020, when transmission of the virus accelerated, and deaths began to accumulate^3^. Several studies have reported the harmful social repercussions of this delayed and conflicted public health response to the pandemic in the United States^3–6^. For example, researchers at the National Center for Disaster Preparedness of Columbia University estimated that 130,000 to 210,000 deaths could have been avoided at the beginning of the pandemic with early policy interventions and a robust federal response^4^.

In this setting, one of the primary lessons public administrators and health researchers have learned from the COVID-19 crisis is the importance of a rapid and coordinated public response that adapts to the explosive dynamics of a pandemic, and the need for informative public health surveillance systems, to prevent future crises^7,8^. Fortunately, the prevalence of electronic health records (EHRs) allows the tracking of general population trends, offering a real-time data-based assessment of the health status of a community. During the pandemic, EHRs have been used to compute accurate individual-level risk scores for predicting death and severe disease after contracting COVID-19^9,10^. These models have helped to prioritize and allocate resources to the most vulnerable individuals, promoting more equitable and efficient distribution. Simultaneously, different groups have created large-scale prediction models to anticipate the course of the pandemic, forecast the spread of the infection, and aid in elaborating health policies that respond to future population trends^11–18^.

However, these models became available only after COVID-19 became a global pandemic and sufficient data was collected to fit the modeling equations reliably. Despite the previous experiments for developing early warning systems, such as Google Flu^19,20^, healthcare systems failed at the most critical task: detecting the pandemic at an early stage and alerting authorities to the imminent pandemic surge before it became a titanic task. The failure to detect the early signs of the impending crisis can be attributed mainly to the absence of tools that proactively track the general population's health with easy-to-interpret results, on a long-term and large-scale basis.

Hence, this study aimed to investigate the use of readily available EHR patient records in detecting the warning signs of approaching health crises. Departing from a multivariate artificial intelligence (AI) model initially developed to predict the risk of high-flow oxygen therapy or mechanical ventilation requirement in COVID-19 patients, we sought to characterize the temporal trends of several population health indicators before and during the pandemic and determine their potential roles as early warning signs of a future health crisis.

## Methods

### Data

The Institutional Review Board of Massachusetts General Hospital approved this single-institution study (protocol number 2021P003232), waiving the informed consent requirement due to its retrospective nature. All procedures were compliant with the Health Insurance Portability and Accountability Act (HIPPA). Data was collected from the emergency department (ED) of Massachusetts General Hospital from January 1, 2019, to August 7, 2020 (the end of the first major COVID-19 wave). A total of 377,694 patient records and 303 features were included in the analysis; patient identifying data was not used, and was removed from the dataset by the authors, thus resulting in de-identified patient records. The features used were different quantitative and categorical variables extracted from our EHR, including but not limited to patient vital signs, laboratory results, and known comorbidities. The outcome selected for modeling was a binary variable indicating whether or not the patient required high-flow oxygen or mechanical ventilation within 48 h of arrival at the ED.

### Features of interest and initial model

For training our models, we intentionally considered only the patient encounters starting in March 2020—when COVID was officially recognized and our hospital experienced a large influx of COVID patients. Many features in the original dataset had missing values (such as lab results, patient history, and vitals), which could not be imputed, thus limiting the choice of machine-learning model. Besides, the large number of original features (303) had to be reduced to avoid overfitting and to retain only the most important factors. Therefore, we started by modeling the outcome with XGBoost (eXtreme Gradient Boosting) AI model, capable of handling missing values and large feature spaces^21^. The XGBoost model (available in Python^22^) was trained on the original data with missing values. Optimal XGBoost parameters (including the number of estimators, tree depth, and L_2_ regularization penalty) were determined with a standard grid search approach and threefold cross-validation to avoid model overfitting. The resulting model yielded an accuracy of 0.81, a good result given the number of missing values and noise.

Once optimized model parameters were determined, the model was run with a model-agnostic permutation variable-importance algorithm^23^, which reduced the original feature set to only the most important 19 features (Table 1). This reduction also enabled us to remove the records with missing values, still retaining most of the original data (236,869 patient records).

**Table 1:** Most important original features identified by XGBoost permutation feature importance algorithm.

Feature label	Feature description
Age	Age of the patient upon entry to the ED
EncounterDiagnoses	Number of diagnoses assigned to the patient after the first encounter
BMI	Body Mass Index
HeartRate	Heart rate at admission
O2Saturation	Oxygen saturation (%) measured by pulse oximetry at admission
RespiratoryRate	Respiratory rate (breaths per minute) at admission
TemperatureF	Patient temperature in Fahrenheit degrees (°F) at admission
ChronicLungDisease	Chronic lung disease (yes/no)
ConnectiveTissueDisorder	Connective tissue disorder (yes/no)
CoronaryArteryDisease	Coronary artery disease (yes/no)
AverageAbsoluteNeutrophils	Average absolute neutrophil count (× 1000/cc)
AveragePercentageMonocytes	Average percentage of monocytes (%)
AveragePercentageNeutrophils	Average percentage of neutrophils (%)
AverageWBC	Average white blood cells count (× 1000/cc)
AveragePercentageLymphocytes	Average percentage of lymphocytes (%)
MCHC	First measured mean corpuscular hemoglobin concentration (g/dL)
FirstPercentageNeutrophils	First measured percentage of neutrophils (%)
LowO2Req	Low oxygen support required in ED (yes/no)
Sex	Patient sex flag (1/0 for male/female)

In parallel with the classical XGBoost approach, we also designed and implemented an exhaustive Boolean rule-learning model to find the best outcome predictors with only up to four feature variables, thus capturing the most important features and the best decision-making logic. Our rule-learning algorithm considered all 4-feature subsets from the original 303 features; non-Boolean features were converted into Boolean by comparing them to their percentile thresholds. For each subset of four features, our algorithm computed all possible 4-variable Boolean truth tables to find the best Boolean rule (as a truth table) to use. Taking advantage of fast vectorized Boolean math and a highly parallelized code run on a 36-core processor, our Boolean rule-learning algorithm discovered the best eight binary rules (*engineered features*), most predictive of the outcome (Table 2). Notably, all original features identified by the rule-learner in Table 2 can also be found in Table 1. Many of the Table 2 features turned out to be very similar to the risk factors reported in other studies^24–26^.

**Table 2:** The most accurate engineered features (short Boolean rules) found by the rule-learning algorithm.

Feature label	Feature rule	Accuracy	Recall
F1	(EncounterDiagnoses > = 2 ***OR*** AverageAbsoluteNeutrophils > = 5) ***AND*** (LowO2Req = *TRUE ****OR*** TemperatureF > = 99)	0.636	0.546
F2	(LowO2Req = *TRUE ****OR*** AverageWBC > = 12) ***AND*** (LowO2Req = *TRUE ****OR*** AveragePercentageMonocytes < 6)	0.632	0.556
F3	(LowO2Req = *TRUE ****OR*** HeartRate > 100) ***AND*** (LowO2Req = *TRUE ****OR*** AveragePercentageNeutrophils > 83)	0.631	0.528
F4	***SUM***(LowO2Req = *TRUE*, AveragePercentageNeutrophils > = 83, CoronaryArteryDisease = *FALSE*) > = 2	0.630	0.589
F5	(LowO2Req = *TRUE ****OR*** AverageWBC > = 12) ***AND*** (LowO2Req = *TRUE ****OR*** AveragePercentageNeutrophils > 83)	0.630	0.576
F6	(LowO2Req = *TRUE ****OR*** AveragePercentageMonocytes < 6) ***AND*** (LowO2Req = *TRUE ****OR*** AveragePercentageLymphocytes < 11)	0.630	0.566
F7	LowO2Req = *TRUE ****AND*** (EncounterDiagnoses > = 2 ***OR*** AverageAbsoluteNeutrophils > = 5)	0.628	0.463
F8	LowO2Req = *TRUE ****AND*** AveragePercentageNeutrophils > 62	0.627	0.462

Note that the prediction outcome in the dataset was significantly unbalanced: patients with a positive outcome (high-flow oxygen or mechanical ventilation requirement within 48 h after ED arrival) corresponded to only 5% of the final data set. As a result, to train XGBoost and Boolean rule models with a balanced dataset, we had to balance the training set by randomly selecting an equal number of patients with positive and negative outcomes. Therefore, both models were trained on 5% of the dataset and tested on the remaining 95%.

As a result, the application of XGBoost and Boolean rule learning models helped us confirm the optimal subset of 19 features, and to remove the missing values present in these features only. For the final ED patient risk model, we were required to use a simple, interpretable, and robust model type, so we chose logistic regression, which became possible after eliminating the records with missing values, as described above. Similar to XGBoost, the logistic regression model was used to predict the probability of supplemental oxygen within 48 h of ED arrival. Quantitative features, Boolean included, were standardized (centered and scaled to unit variance) before analysis.

### Pre-pandemic trends and early pandemic signs

Our model was initially developed to predict the risk of COVID-19 patients requiring high-flow oxygen supplementation or mechanical ventilation based on data from the initial pandemic wave (March to August 2020). However, after verifying that the model tracked well with the COVID-19 counts during the pandemic, we decided to perform a "post hoc" analysis, running the model "back in time." To account for any seasonal effect, we set the pre-pandemic date range to January 2019. We wanted to check whether (a) the high-flow oxygen or mechanical ventilation requirement model, initially applied during the pandemic, would detect any alarming signal during the pre-pandemic months, and (b) whether the trends detected by the model could also be recognized in the original model features, free of specific model/pandemic context. To do so, we decided to assess the temporal trends of each feature included in the final model, and determine if any relevant change occurred before the first cases were officially reported and the pandemic emergency was declared in the United States.

## Results

The logistic regression model for predicting the need for oxygen supplementation within 48 h after ED arrival in high-risk COVID-19 patients was trained on the March-August 2020 data, as the time when the COVID pandemic was officially declared in the US. The model had an AUC ("area under the curve") value of 0.72, with an accuracy of 74%. Given the high variability and noise of the real-life data, we considered these values to be satisfactory and proceeded to analyze the temporal trends of the model and its features.

### Temporal trends: model

**Figure 1:**
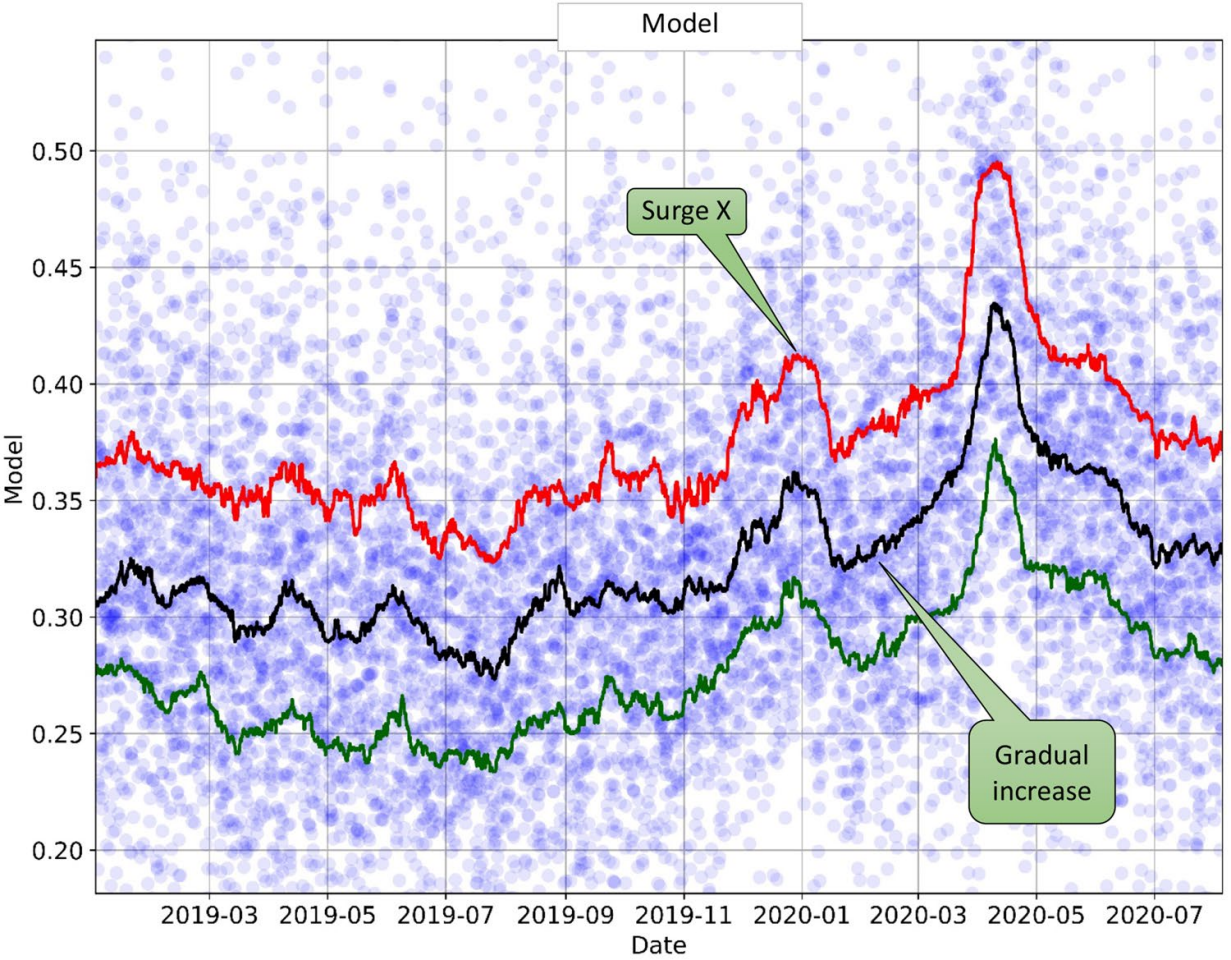
Predicted fraction of patients requiring oxygen within 48 h of ED arrival plotted over time (from January 2019–August 2020). The circles represent the original data averaged over 6-h intervals (with 3-h increments). Lines represent the percentile curves: the 25th percentile (green line), median (black line), and 75th percentile (red line). The percentile trend curves exhibit temporal patterns despite the large data variability and noise.

To detect any temporal changes in the population health dynamics, the model results and the included features were analyzed by visualizing their trends over time. This approach is shown in Fig. 1, using the original model outcome as an example. In this graph, each sample point was created by averaging the model values for the individual ED patients over a 6-h interval, taken at 3-h increments, to reflect the changing health dynamics of the patients arriving at the ED. To visualize these population health trends better, we added three moving percentile curves: the 25th percentile (lower line), median (middle line), and 75th percentile (upper line), applied to the points in the chart over a 1-week moving window. As a result, this graph allowed the visualization of the distribution and variability of the original data and their temporal trends (from January 2019 to August 2020).

Note that despite the noise in the individual patient measurements, the population-level model tracked very well with the overall increase of COVID-19 patients in the ED during the peak of the pandemic's first wave (April to May 2020, Fig. 2, COVID trend on top vs. model trend in the bottom). Interestingly, the model showed a gradual increase in the high-risk patient population even before the pandemic was officially declared in March 2020. Additionally, we noted a significant and unusual pre-pandemic surge in the number of high-risk patients that began in late 2019, peaked in early 2020, and converged with the gradual increase of cases associated with the initial wave of the COVID-19 pandemic (see "Surge X" in Fig. 1). This Surge X pattern in the pandemic model trend was not present the year before the pandemic (late 2018-early 2019), suggesting that an additional variable was affecting the trends during this period and a non-seasonal cause was responsible for this phenomenon.

**Figure 2:**
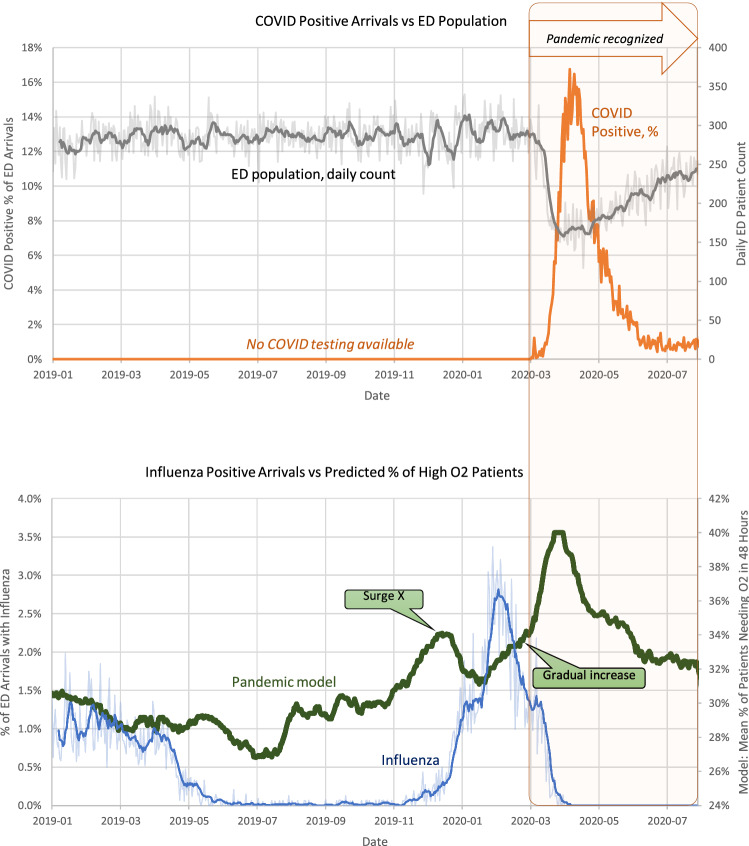
Top: Percentage of ED patients who had positive COVID-19 test results versus the daily ED population count; regular COVID-19 tests became available in March 2020. Bottom: Percentage of ED arrivals who received a positive influenza test versus our pandemic model trend. Darker lines show 1-week moving averages, and lighter background lines—the actual daily values.

Considering that Surge X could have been explained by any acute respiratory illness, including an outbreak of seasonal influenza or early COVID-19 cases, we decided to investigate the overall contribution of influenza during the same period. Notably, we found that while the proportion of ED patients who tested positive for influenza increased right before the first COVID-19 pandemic peak (Fig. 2, bottom), influenza could not explain Surge X. As seen in Fig. 2, Surge X occurred before the influenza cases started to increase, and its slope decreased as the number of influenza cases started to peak.

Thus, influenza alone does not seem sufficient to explain the late-2019 Surge X pattern in the pandemic model trend or the gradual increase in cases that took place as the COVID-19 pandemic started. Furthermore, we confirmed that this pattern was not present during the 2018–2019 period, indicating that "Surge X" trend does not represent a predictable seasonal increase in high-risk patients. While it is not possible to retrospectively asseverate that Surge X represents an early surge in COVID-19 cases, it may be possible that several detectable changes in the population health dynamics preceded the pandemic and the exponential increase in the number of cases that took place during the first pandemic wave in spring 2020. Furthermore, after our analysis was completed, newer reports based on blood sample analysis confirmed the presence of COVID-19 cases in our state in late 2019, supporting our hypothesis that the trend discovered in the EHR was indicative of early signs of the impending pandemic^27^.

### Temporal trends: individual features

**Figure 3:**
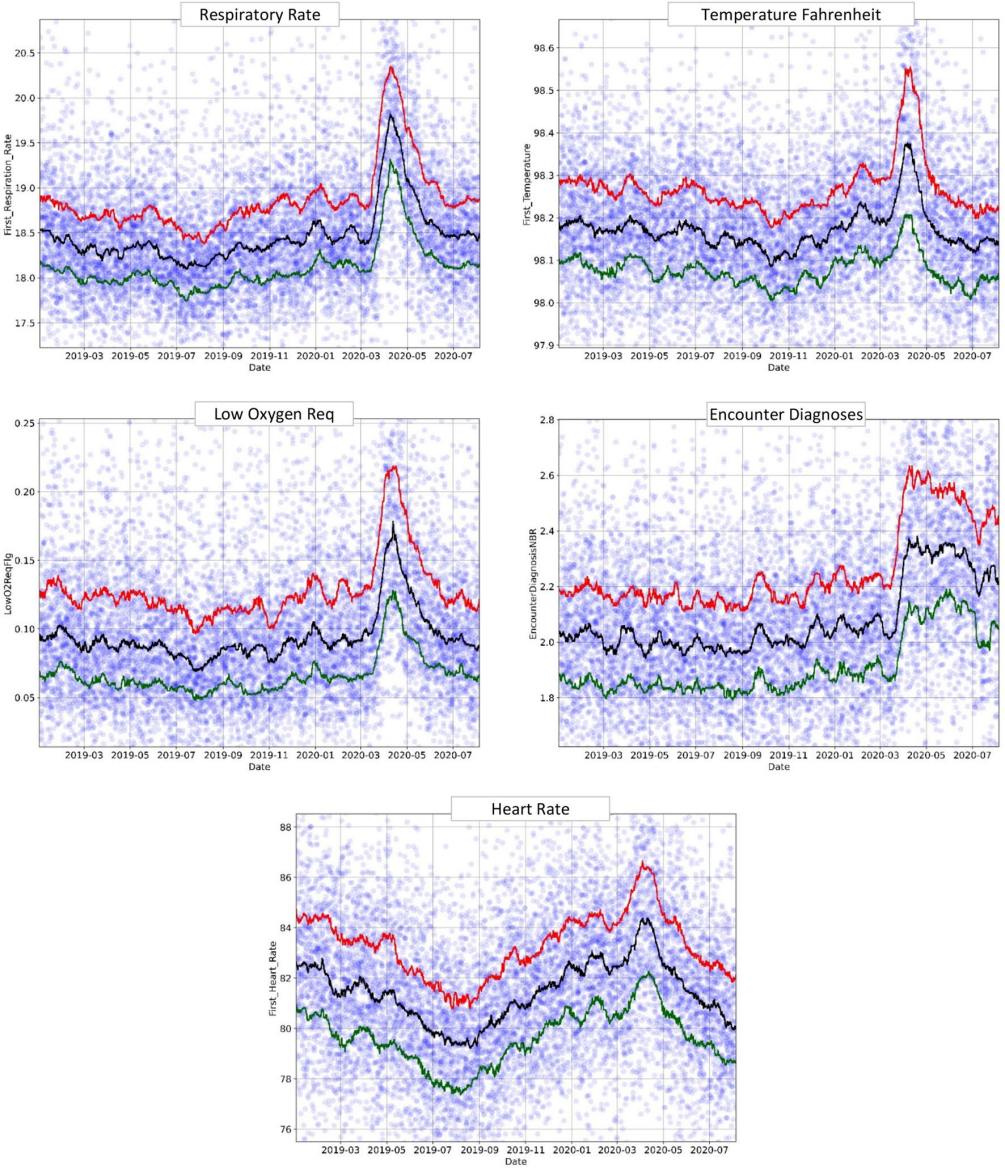
Respiratory rate, temperature, low O_2_ requirement, number of diagnoses at first encounter, and heart rate model features plotted through time (January 2019–August 2020). The circles represent the original data averaged over 6-h intervals, and the lines-moving percentile curves for 25%, 50%, and 75%.

To confirm that the unusual changes in the population health detected by the model were not produced by a specific model bias or training artifact, we verified whether the individual model's features, free of any modeling context, also revealed the same changes. To do so, we plotted the temporal trends of these features in the same way as the overall model. From these plots, we identified five features (respiratory rate, temperature, low oxygen requirement, number of encounter diagnoses, and heart rate) that showed a solid and rapid increase in the ED population in early March 2020 (COVID-19 outbreak) and a decline in May 2020 (end of the first COVID-19 wave), as shown in Fig. 3. What was particularly important is that many of these features presented the same "Surge X" pattern, thus proving that it was independent of our initial model.

There were also a few deviations from this general trend. First, the EncounterDiagnoses feature showed only a mild surge in late 2019 but a sharp spike in March 2020. This behavior probably reflects the diagnostic and therapeutic challenges associated with the first COVID-19 cases in the pandemic setting (Fig. 3). Second, the heart rate had its own unique superimposed trend. Without considering the effects of the pandemic, as reported previously, the heart rate usually follows a seasonal trend, reaching a maximum in January and then declining steadily to a minimum in July^28^. This heart rate population behavior was seen in our data as well. However, we found an unusual surge in heart rate at the end of February 2020, most likely corresponding to the influx of pandemic patients. Thus, even though the heart rate shows its distinctive seasonal pattern, it was still possible to observe the pandemic effects on the ED population.

**Figure 4:**
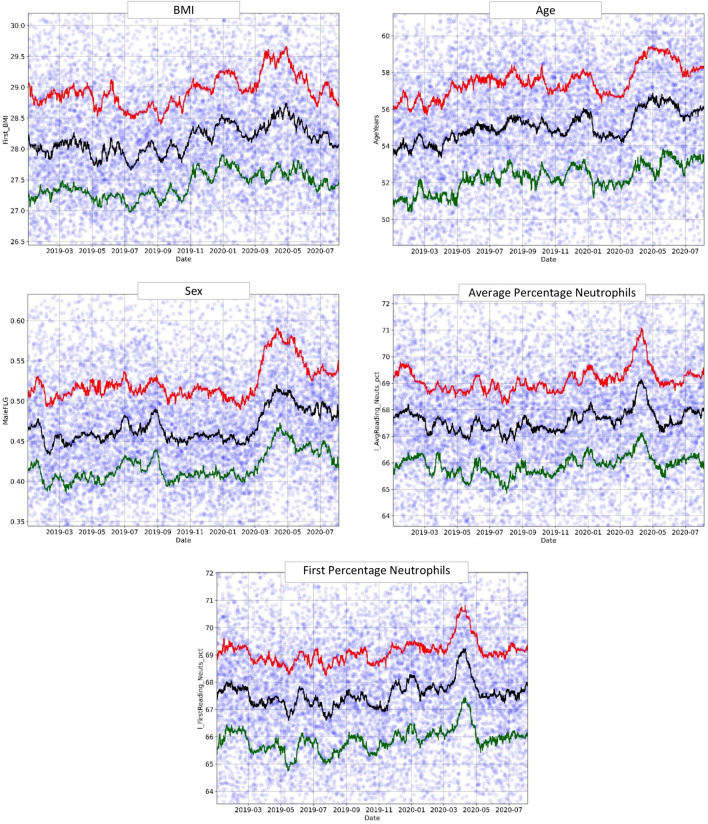
BMI, age, sex, and neutrophil percentage model features plotted over time (January 2019–August 2020). The circles represent the original data averaged over 6-h intervals, and the lines-moving percentile curves for 25%, 50%, and 75%.

Other features, including BMI, age, sex, and neutrophil percentage (average and at first encounter), exhibited broader positive changes and more noise. Nevertheless, these changes followed the pandemic timeline and displayed the pre-pandemic "Surge X" (Fig. 4). This is particularly important because these features are usually constant in a specific population and do not follow seasonal trends. However, in this case, they began to change as the pandemic approached. This phenomenon strengthens our central hypothesis: before the pandemic was officially declared (March 2020), the characteristics of the ED population started to exhibit dramatic changes and adopted a "pandemic" profile^23^.

**Figure 5:**
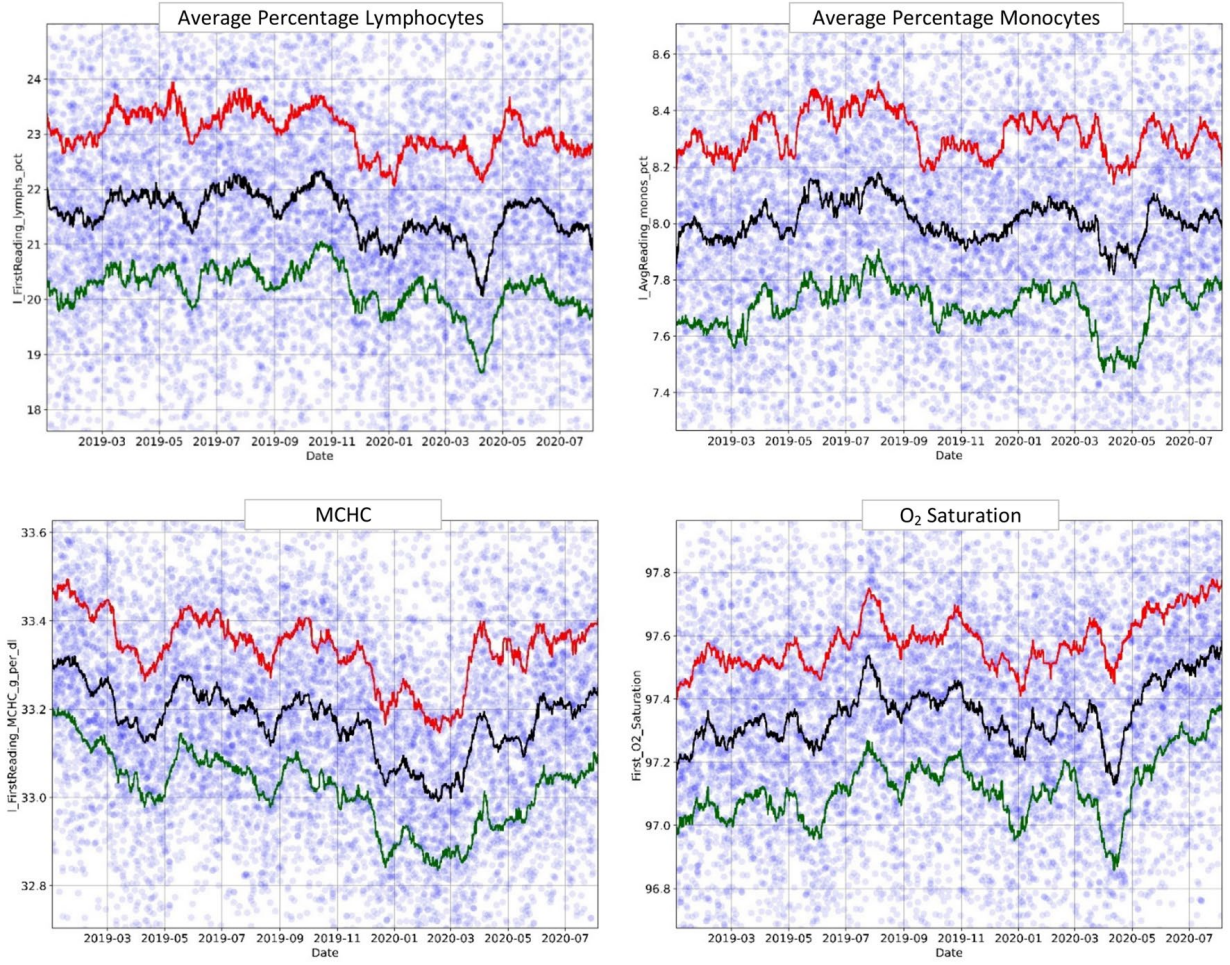
Decreasing trends: lymphocyte and monocyte percentages, MCHC, and O_2_ saturation over time (January 2019–August 2020). The circles represent the original data averaged over 6-h intervals, and the lines-moving percentile curves for 25%, 50%, and 75%.

Additionally, some features (lymphocyte and monocyte percentages, MCHC, and oxygen saturation) displayed an inverse relation with the number of COVID-19 patients, including a pre-pandemic decrease equivalent to "Surge X" (Fig. 5). This inverse relationship was expected based on the usual clinical behavior of COVID-19 patients.

Finally, several other features were analyzed, including the presence of chronic lung disease, connective tissue disorder, coronary artery disease, average white blood cell count, and average absolute neutrophil count. These features showed different surges over time, but none led to a clear and dominant trend.

### Temporal trends: engineered features

**Figure 6:**
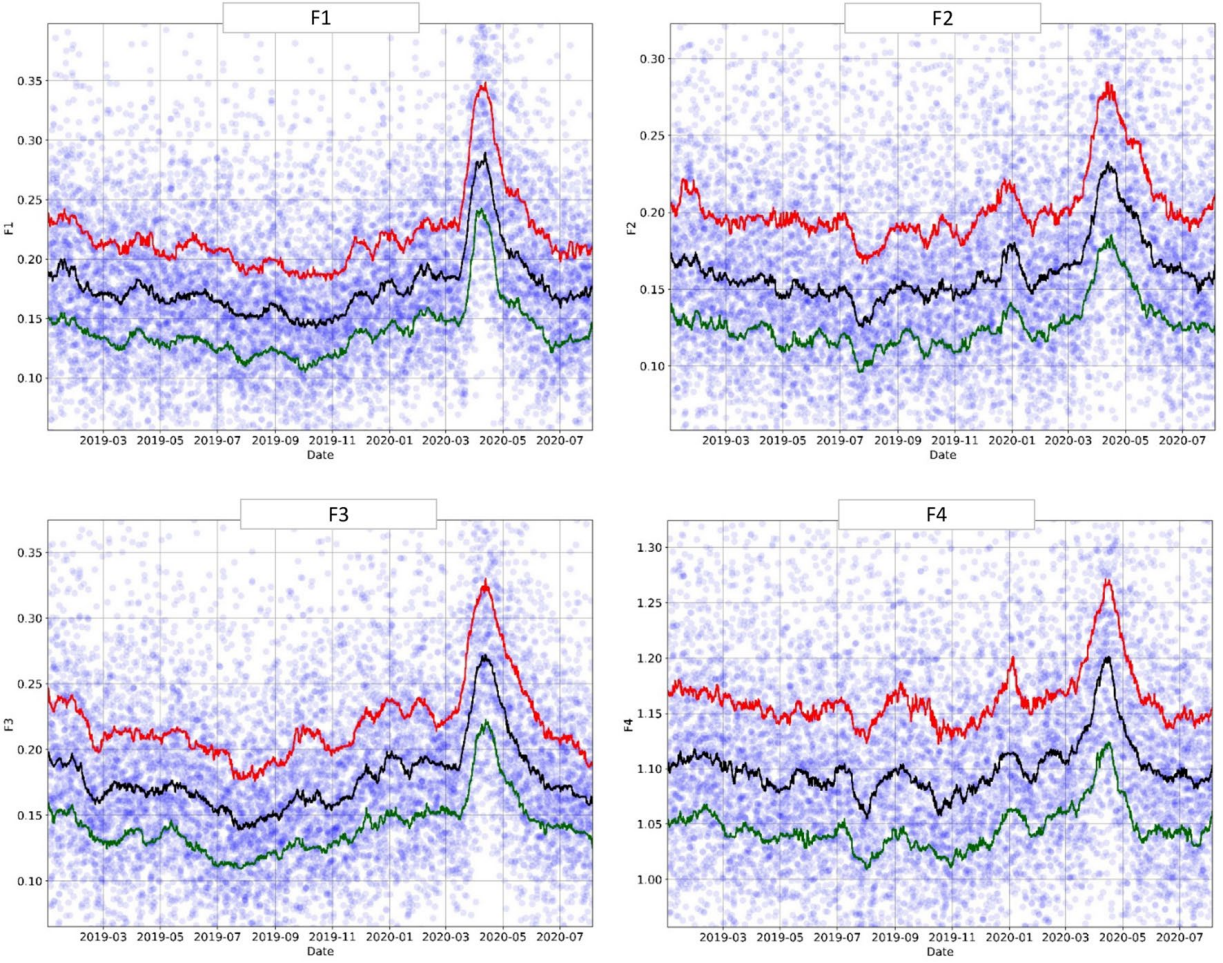
Engineered feature (F1-F4): trends over time (January 2019–August 2020). The circles represent the original data averaged over 6-h intervals, and the lines—moving percentile curves for 25%, 50%, and 75%.

**Figure 7:**
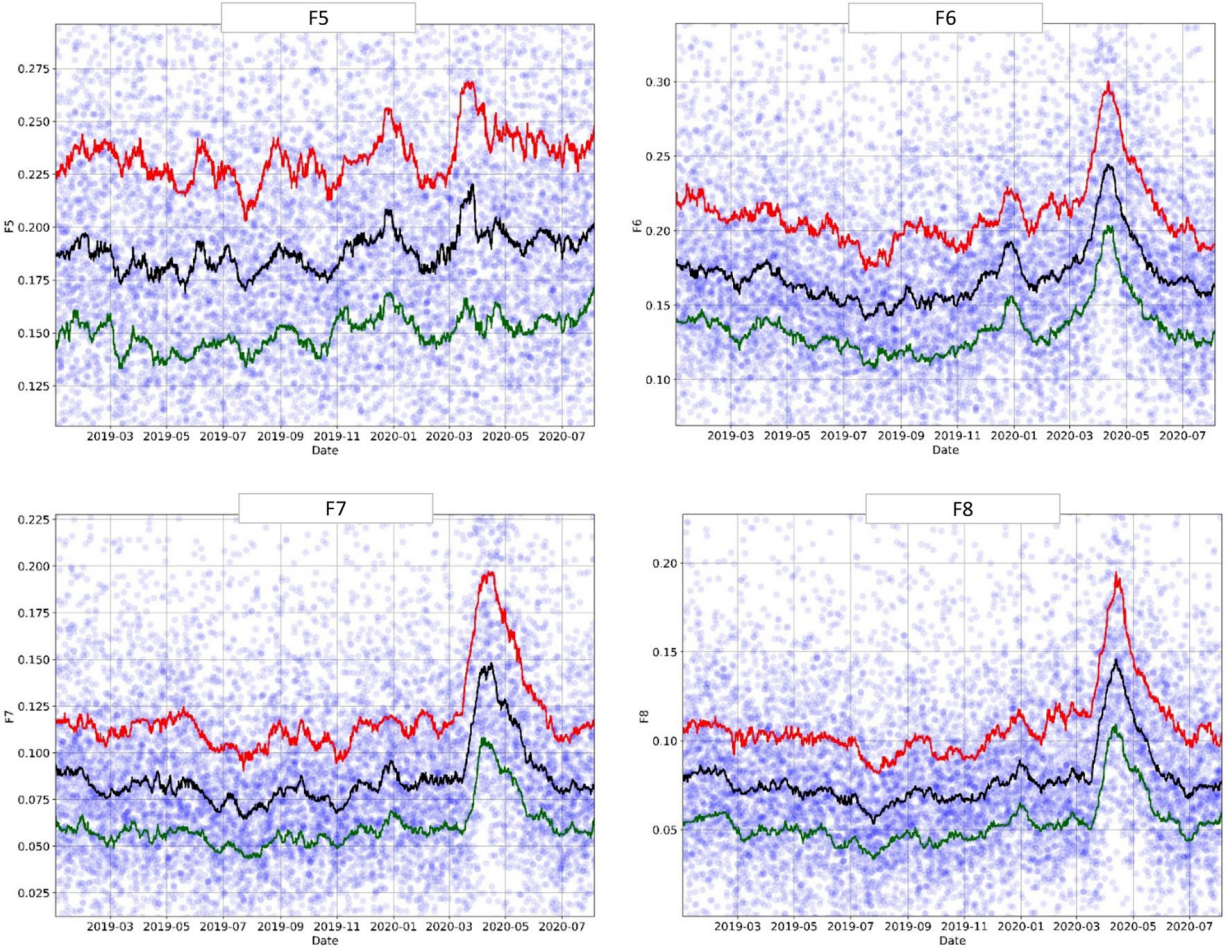
Engineered feature (F5-F8): trends over time (January 2019–August 2020). The circles represent the original data averaged over 6-h intervals, and the lines—moving percentile curves for 25%, 50%, and 75%.

The engineered features (Table 2), produced by the exhaustive binary rule search, showed the same sudden increase in the number of pandemic patients starting in March 2020, peaking in April 2020, and then decreasing in May 2020 (end of the first pandemic wave), as can be seen in Figs. 6 and 7. Moreover, most of these features (F2, F4, F6, F7, F8) showed a "Surge X" peak in early January 2020. In contrast, other features (F1, F3) exhibited a steady increase beginning in November 2019 and leading to the COVID-19 outbreak in March 2020. F5 was less sensitive than the other engineered features.

Note that these features were found independently of the original machine-learning model and therefore did not depend on the model selection or training. Instead, the engineered features provided us with more interpretable and targeted metrics of the overall patient population health behavior, which proved to be consistent with pandemic trends and the output from the more complex model. This demonstrates that one can use routinely collected EHR metrics to develop very simple rules, serving as sensitive markers of current population health, and capturing the "signal above the noise".

## Discussion

### Early signs of health crises

Major changes in the population's health cannot happen overnight—instead, they take significant time to develop and spread. In this single-institution retrospective study, we studied the temporal trends of several population health indicators before and during the COVID-19 pandemic to determine their potential role as early warning signs of a future health crisis. Departing from an AI model initially designed to predict the need for high-flow oxygen or mechanical ventilation in COVID-19 patients, we analyzed the individual variables included in the model and found that many of them began to exhibit alarming patterns several weeks before the pandemic was officially recognized and declared. This important result suggests that significant changes in population health trends may serve as early warnings of future crises, implying the need for developing patient health surveillance systems. To our knowledge, this is the first study to explore this issue and offer a practical approach for populational health surveillance that can significantly improve our response to future health crises.

It was particularly reassuring that during the preparation of this publication, a growing volume of new, independent evidence supported our approach and findings. Several studies had suggested that the SARS-CoV-2 was already circulating in China from mid-October to mid-November of 2019, a few months before the first cases were officially reported^29–31^. Similarly, new studies had shown serological evidence of the virus in samples of patients seeking medical care in the United States and Europe as early as December 2019^27,30–32^, including at least one patient located in our hospital area. The latter case was dated January 8, 2020, thus falling on the peak of the "Surge X" curve, identified by our approach. This alignment between our data-driven method and the independent blood sample research suggests that the virus was circulating before the first cases were officially recognized, confirming the validity of our approach and results^30^.

### Building an efficient population health surveillance system

The COVID-19 global pandemic has caused unprecedented worldwide changes in healthcare delivery^33–35^. While containment and mitigation approaches have been intensified, the exponential and progressive increase in the number of cases has overwhelmed health systems globally^36,37^, highlighting the need for anticipation and prediction to be the base of an efficient response system for a future pandemic scenario. Response strategies for population-wide outbreaks currently adopt a passive and "a posteriori" approach. With our approach, real-time health surveillance systems that monitor population health deviations offer a much more efficient and proactive alternative.

Our results demonstrate that several clinical features started to display differential and unusual trends a few weeks before the pandemic was declared worldwide. At a population level, the respiratory rate, temperature, low oxygen saturation, number of diagnoses during the first encounter, and heart rate demonstrated observable and traceable changes *eight weeks before* the outbreak and public health emergency declaration. These trends followed the surge of cases during the first pandemic wave. This is particularly important because, unlike the original model, the analyzed individual features we considered were completely free of any specific model or "risk factor" assumptions. Yet they started to deviate from their usual patterns weeks before medical professionals became aware of a potential issue.

**Figure 8:**
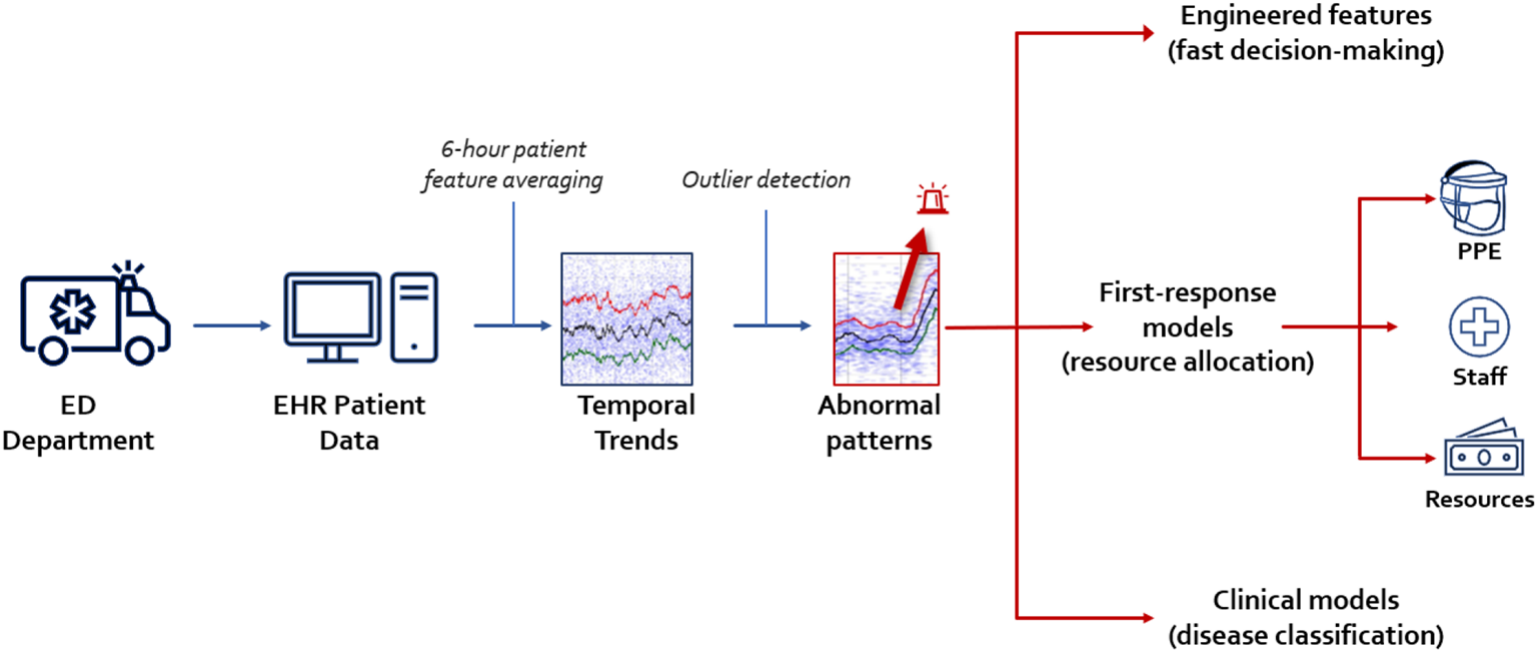
Population surveillance model: major components.

Importantly, these early warning features came from a standard EHR system, where features are routinely recorded in a real-time fashion for all patients arriving at the hospital. As a result, the widespread adoption of EHR systems and patient databases makes population health surveillance an accessible and effective way of anticipating a future health crisis. Based on the analysis we have developed, we recommend the following steps to implement an efficient population health surveillance system (Fig. 8):

- *Computing real-time population health features* (vitals and major laboratory studies). As described in our study, we achieved this by averaging the individual patient features every 6 h. A longer window may be used to ensure sufficient samples.
- *Visualizing current feature trends* to display the moving median and low/upper bounds (25th and 75th percentile) of the current feature distribution (Fig. 1). Visually displaying the temporal behavior of a feature helps capture non-trivial patterns in the feature evolution, similar to the surge patterns we presented. Particular emphasis should be placed on tracking those features that cannot change abruptly (such as BMI, age, or sex) —any unusual deviations in them mean that these features evolve into triggered risk factors, changing the distribution of the people seeking urgent care.
- *Evaluating standard statistical controls* to detect whether a particular feature deviates from its expected value range. Their controls may depend on a particular location, subpopulation, and expected behavior, so each healthcare institution should characterize its own trends and create a baseline for subsequent analysis. This initial characterization can be done with a standard control chart approach. To account for seasonal changes, comparisons should be performed between the same period in different years.

In addition, we also suggest developing a second, advanced layer to support timely decision-making, including (Fig. 8):

- *Engineered features,* fine-tuned into specific types of outbreaks. Similar to our engineered feature example, these can better identify particular patient conditions for which they were developed: flu, SARS, other infectious diseases, and more. These short conditional expressions, which can be found with rule-learning algorithms based on 3–4 original features and thresholds, can be easily understood and applied by humans, providing efficient early diagnostic markers.
- *Clinical decision-making models* are based on the above and used as a preemptive response to emerging outbreaks. For instance, if any of the features begin to exhibit alarming deviation from the normal trend, resource allocation should be prioritized to respond to the emerging situation, and additional tests need to be implemented to identify and characterize the nature of the new crisis.

Using short engineered-features becomes particularly important when building AI models is not feasible due to limited resources and time.

### Limitations and next steps

Our study is limited by its retrospective and single-center design leading to a noncontrolled study population. However, the number of patients included in our study supported our goal and demonstrated our hypothesis, making our findings generalizable to larger populations. In addition, while there is no way to objectively and retrospectively demonstrate that "Surge X" was indeed caused by a pre-pandemic surge of COVID-19 cases, the most recent studies that demonstrated serological evidence of the infection in early 2020 support our findings and conclusions.

While practical limitations exist, we would like to emphasize that our primary approach is based on the gradual spread of pandemics—the principle widely recognized by practitioners, researchers, and pandemic models (such as SEIR). This principle, combined with the critical concept of early detection (adapted long ago in individual patient screening), extends very naturally to a broader population level, justifying our approach. Consequently, we believe that more research needs to be done in the area of population health screening and early alarms identified from the key population data, to understand their nature better and forecast their impact.

## Conclusion

Our work demonstrates how the time-windowed aggregation of individual patient metrics, routinely collected in hospital emergency departments, can lead to robust markers of approaching health crises. This finding, confirmed by recent blood test research, provides a strong foundation for implementing robust population surveillance systems, detecting abnormal development several weeks in advance.

This surveillance, driven by the data already available in EHR, does not require prior knowledge of the emerging ailment and, therefore, could be applied to various diseases and in several contexts. Moreover, its baseline outlier detection should be further complemented with the development of proactive response protocols, prescribing which immediate steps need to be taken should certain features surpass their standard thresholds. These steps should also cover a wide range of actions, from further investigating the clinical nature and possible source of the change (including EHR data on patient locations), to assigning additional resources (beds, PPEs, medications).

Acting promptly should become the primary approach to preventing the losses brought about by population-wide health crises.

## Data Availability

The data used in the current study is available from the corresponding author on reasonable request.
